# Utility of Environmental Complexity as a Predictor of Alzheimer’s Disease Diagnosis: A Big-Data Machine Learning Approach

**DOI:** 10.14283/jpad.2023.18

**Published:** 2023

**Authors:** M. Yuan, K.M. Kennedy

**Affiliations:** 1. Department of Geospatial Information Sciences, School of Economic, Political and Policy Sciences, The University of Texas at Dallas, Richardson, TX USA;; 2. Department of Psychology, School of Behavioral and Brain Sciences, Center for Vital Longevity, The University of Texas at Dallas, Dallas, TX, USA

**Keywords:** Alzheimer’s disease, neural network modelling, environmental complexity, cognitive map, geospatial mapping

## Abstract

**BACKGROUND:**

Rural-urban differences and spatial navigation deficits have received much attention in Alzheimer’s Disease research. While individual environmental and neighborhood factors have been independently investigated, their integrative, multifactorial effects on Alzheimer’s diagnosis have not. Here we explore this “environmental complexity” for predictive power in classifying Alzheimer’s from cognitively-normal status.

**METHODS:**

We utilized data from the National Alzheimer’s Coordinating Center (NACC) uniform data set containing annual visits since 2005 and selected individuals with multiple visits and who remained in their zipcode (N = 22,553). We georeferenced each subject with 3-digit zipcodes of their residences since entering the program. We calculated environmental complexity measures using geospatial tools from street networks and landmarks for spatial navigation in subjects’ zipcode zones. Zipcode zones were grouped into two cognitive classes (Cognitively-Normal and Alzheimer’s-inclined) based on the ratios of AD and dementia subjects to all subjects in an individual zipcode zone. We randomly selected 80% of the data to train a neural network classifier model on environmental complexity measures to predict the cognitive class for each zone, controlling for salient demographic variables. The remaining 20% served as the test set for performance evaluation.

**RESULTS:**

Our proposed model reached excellent classification ability on the testing data: 83.87% accuracy, 95.23% precision, 83.33% recall, and 0.8889 F1-score (F1-score=1 for perfect prediction). The most salient features of “Alzheimer’s-inclined” zipcode zones included longer street-length average, higher circuity, and slightly fewer points of interest. Most “cognitively-normal” zipcode zones appeared in or near urban areas with high environmental complexity measures.

**CONCLUSION:**

Environmental complexity, reflected in frequency and density of street networks and landmarks features, predicted with high precision the cognitive status of 3-digit zipcode zones based on the etiologic diagnoses and observed cognitive impairment of NACC subjects residing in these zones. The zipcode zones vary widely in size (1.6 km^2^ to 35,241 km^2^), and large zipcode zones suffer high spatial heterogeneity. Other proven AD risk factors, such as PM2.5, disperse across zones, and so do individual’s activities, leading to spatial uncertainty. Nevertheless, the model classifies diagnosis well, establishing the need for prospective experiments to quantify effects of environmental complexity on Alzheimer’s development.

## Introduction

**T**he US Center for Disease Control and Prevention’s Healthy Brain Initiative and the Alzheimer’s Association (3–5) list ten (10) warning signs of Alzheimer’s Disease (AD) (1, 2). Empirical and clinical studies support the efficiency of these warning signs on AD screening (3–8). Four of the ten early symptoms relate to spatial dysfunctions, including difficulty completing familiar tasks at home, at work, or at leisure (e.g., trouble driving to familiar locations), confusion with time or place (e.g., forgetting where they are or how they got there), trouble understanding visual images and spatial relations (e.g., trouble judging distance), and misplacing things and losing the ability to retrace steps (e.g., putting things in illogical places and unable to go back over the steps to find them again). These spatial dysfunctions frustrate and endanger AD patients in their spatial cognitive activities daily. Connecting spatial cognitive activities to AD development may present opportunities for non-pharmaceutical interventions to delay or slow AD progression.

Early neuropathology of AD occurs in the entorhinal cortex (9), leading to spatial navigation impairment that differentiates patients with mild cognitive impairment (MCI) and AD from cognitively healthy aging adults (10, 11). Specifically, MCI and AD patients experience declining abilities for allocentric navigation with external cues or landmarks. Allocentric navigation reckons directions relative to the Earth, independent of the navigator’s orientation. A common example is navigating with a map. Even without a physical map, one can develop a cognitive spatial map (a.k.a. mental map) as an internal representation of the environment with geospatial features independent of one’s current location or orientation. A system of spatial cells in the hippocampal formation subserves cognitive map building. Place cells fire at specific locations in specific environments and vary the output signals with the engaging behavior. Grid cells and head-direction cells provide universal metrics for mapping positions and directions in all environments. Many studies showed that spatial maps in the brain reflect the complexity of our nested and dynamic environments (12). Multiple cognitive spatial maps and sub-maps provide reference frames for subdivisions of our environments, each of which includes a collection of landmarks, geometric relationships, and additional non-geometric properties (13). The fragment fitting theory posits that the brain rotates and assembles selected sub-maps to form a local map during navigation (14, 15). As such, a complex urban environment may offer daily opportunities for building and operating mental maps to stimulate these spatial cells. Will these mental exercises routinely excite spatial cells and subsequently delay AD onset or slow down AD progression?

There is hopeful evidence for the excitatory effects of spatial cognitive activities on neural activation and neuroplasticity. While studies on grid cells so far have been limited to mice, fMRI research on human subjects reveals that recalling location information triggers greater anterior hippocampal activation, whereas retrieving spatial relation excites greater activation in the posterior hippocampus (16). The finding of anterior-posterior hippocampal differential in human brains is consistent with grid-cell excitability and the gradient of increasing spatial granularity of grid maps along the ventral-dorsal entorhinal axis in rats (17). Furthermore, studies on London taxi drivers suggest that recalling landmarks and spatial layouts activates the right hippocampus (18) and acquiring the complex spatial structure of London’s streets and landmarks in 3–4 years of taxi-driver training drives structural brain changes with more gray matter volume in the posterior hippocampus and less gray matter in the anterior hippocampus compared to non-taxi drivers (19) and even bus drivers (14). Findings of structural brain changes in London taxi drivers (18) and spatial information retrieval (16) support the potential excitatory effects.

This research expands upon key findings from previous studies on the support of hippocampal formation on allocentric cognition and AD pathology. Grid cells in the medial entorhinal cortex support path integration using self-motion signals of landmarks to estimate travel distance, direction, and self-location, which are critical to successful allocentric navigation. The spatial periodicity of grid-cell firing fields forms the brain’s metric coordinate system for allocentric navigation (20). Excitatory neuronal loss and grid-cell dysfunction, including destabilized grid fields and reduced firing rates, were associated with tau pathology (21) that drives a slowly progressive retrograde neurodegeneration with accumulating neurofibrillary tangles in the somatodendritic compartment of entorhinal cortex neuron in early AD (22). Although AD etiology remains uncertain, the disease has strong associations with neurodegeneration that leads to a declination of spatial cognitive capabilities and topographic disorientation when navigating in familiar places.

As such, we hypothesize that regular mental exercises on cognitive map building can evoke excitatory effects on space-cell firings and, therefore, can delay AD onset and decelerate AD progression. Taking a data-driven approach, this study develops a machine-learning model with existing large-scale AD data (detailed in Methods section) to complement existing experimental and clinical studies on the effects of environmental, socio-economical, and behavioral factors on the disparities in dementia and AD (23–25). In epidemiology, the exposome concept complements the genome by considering one’s lifelong exposure to internal, general external, and specific external factors and their change over time (26). Likewise, AD exposome considers endogenous (i.e., internal, as individual biomes, fat depots, or traumatic brain injury), exogenous macro-level (i.e., general external factors, such as rural vs. urban environment, pollutants, socioeconomic status), exogenous individual-level (i.e., specific external factors, such as diet, infection, etc.) factors as well as interactions among these factors (23). Evidence from these studies thus far implicates air pollution, neighborhoods area deprivation index, education, and income, for example, with an increased odds of AD neuropathology. None of these AD-exposome studies, however, examines the potential influence of environmental complexity on Alzheimer’s Disease via the mediation of spatial cognition and navigation despite the strong connections of spatial cognition and navigation to major categories of spatial cells in our hippocampal formation (27, 28) and furthermore to early cognitive symptoms of MCI and AD (10).

**Figure 1.**
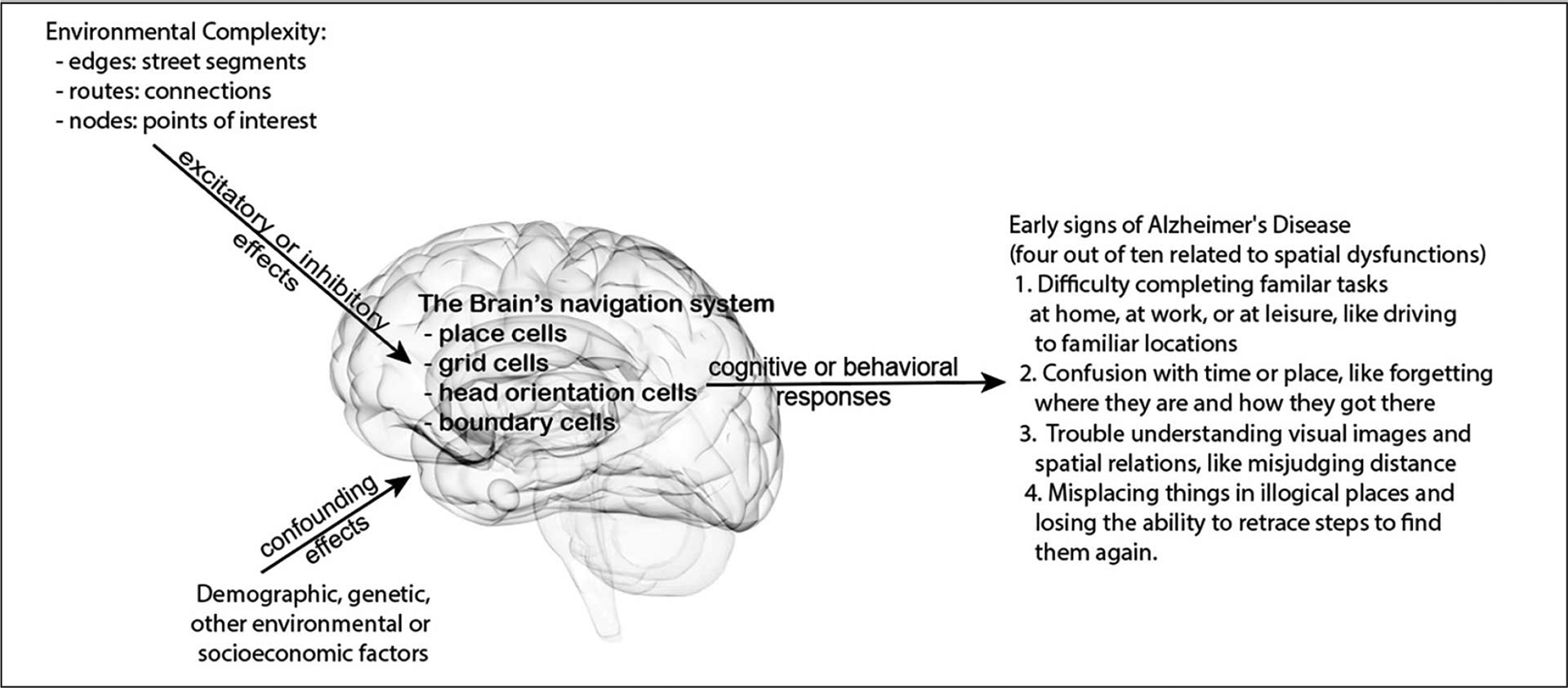
Conceptual model hypothesis connecting the potential effects of environmental complexity on spatial cognition and behaviors to some early cognitive signs of Alzheimer’s disease

In this exploratory retrospective study, we consider these connections of environmental complexity, allocentric navigation, cognitive maps, via their neurological substrates, on AD development in the conceptual model presented in Figure 1. Will a more complex environment impose a higher demand on cognitive map building for allocentric navigation? If so, people who live in a more complex environment likely have more opportunities to frequent mental exercises of building and retrieving cognitive maps during daily commutes and routine errands, leading to excitatory neuronal engagement effects that impede AD development. We posit that a greater reliance on allocentric spatial cognition is related to lowered AD risk and higher environmental complexity enforces cognitive map building for allocentric navigation routinely. Therefore, when controlling other salient demographic, environmental and socioeconomic factors, we expect fewer mild cognitive impairment (MCI) and fewer AD patients in more complex environments. Using data from the National Alzheimer’s Coordinating Center (NACC) and geospatial data, we analyze environmental complexity and MCI/AD population in subjects’ residential 3-digit zipcode zones to examine whether 3-digit zipcode zones of higher environmental complexity relate to lower MCI/AD occurrences in proportion to the participants dwelling in each zipcode zone.

## Methods

The project acquired data from three independent sources to account for Alzheimer’s subjects, road networks, and landmarks/points of interest, described respectively, below. Lynch’s seminal book titled The Image of the City (29) highlighted five elements that form a city’s image: paths, edges, districts, nodes, and landmarks, and stressed the need to go beyond single elements to emphasize their patterning into a complex visual whole (30). The five elements constitute a city’s legibility characterizing the degree to which the urban environment feeds into the creation of mental maps through navigating the city. We followed Lynch’s thesis and adopted the five elements to represent the construct of environmental complexity in cognitive map building. Data for paths, edges, and nodes come from measures of street networks. Points of interest (POI) capture landmarks with various geographic features, physical structures, buildings, shops, and districts such as shopping malls, schools, industrial establishments, government facilities, etc. We examined the proportions of cognitively normal, dementia and AD subjects in each zipcode zone and the measures of zipcode zones’ environmental complexity.

### NACC subjects

We used the Uniform Data Set (UDS) from the National Alzheimer’s Coordinating Center (NACC) with annual visits of subjects since 2005. NACC collected the subjects from the 37 National Institute of Aging Alzheimer’s Disease Research Centers (ADRCs) across the continental US. Each annual UDS visit asks clinicians to complete 16 data-collection forms on various topics regarding demographics, neurological examination findings, and diagnosis with cognitive status ranging from normal cognition to probable AD. Detailed demographic data are presented with cognitive statistics in Results section.

NACC subjects are referral-based or volunteer cases. Therefore, they are not a statistically based sample of the US population nor can be used to estimate the prevalence or incidence of dementia subtypes in the general population. Therefore, we calculated the ratios of normal, mild cognitive impairment (MCI), and AD subjects in the smallest geographic unit available for NACC data (i.e., 3-digit zipcode zones) as relative normalized measures. In the US, 3-digit zipcode zones represent postal areas for mail sorting and processing, and their area sizes vary with population distributions: smaller in urban and larger in rural areas. Most popular are 5-digit zipcode zones for mail delivery. Concerns with privacy and confidentiality often limit public release of medical data to 3-digit zipcode zones.

Regarding sample selection, there were N = 37,718 total subjects in the NACC UDS data released in November 2020. Of these participants, N = 25,502 had records of multiple annual visits; and among those, N = 22,553 subjects had multiple visits and remained living in the same 3-digit zipcode zones throughout their visits. Using the 2017 geography files repositories at the US Census Bureau, we retrieved geographical boundary files for the zipcode zones of the selected 22,553 NACC subjects. Many of these zipcode zones contained fewer than 10 NACC subjects; we only retained zipcode zones containing 30 or more NACC subjects for statistical analysis (n = 154 zones). Most NACC subjects with multiple visits in the same zipcode zones were included in the study as can be seen in Table 1; however, the selection process removed more subjects with AD from further analysis from the NACC dataset, which might weaken the signals that our research attempted to identify. Nevertheless, this conservative decision to remove these subjects avoided zipcode zones with small samples that could skew the statistics. Furthermore, participants might have AD in other zipcode zones and later moved to the zipcode zones recorded in the NACC data. As such, we used population percentages of participants in cognitive categories in each zipcode zone to analyze relative patterns rather than absolute counts.

### Network measures

The selected NACC subjects resided across the continental United States. Lynch’s survey approach to quantify a city is impractical (i.e., as Lynch and Banais (30) did) for this nationwide study. Instead, we integrated Lynch’s concepts of city navigability with the recent advances on urban analytics to capture network complexity. Boeing (31–34) developed 43 network measures to comprehensively quantify the geometric, topologic, and positional characteristics of road networks for each census block across the continental United States. Using Open Street Map (OSM at https://www.openstreetmap.org), we calculated 13 network measures related to city navigability (e.g., edges, nodes, and connectivity). Correlation analysis showed several of these measures were highly correlated, with the absolute value of Pearson’s r > 0.70, which implied that they quantified the same network complexity. To avoid duplicated measures that could bias the findings, we selected the four least correlated network measures (see Table 2) with high correlations with the unselected measures to quantify edges, nodes, topology and path options in networks within each of the 154 3-digit zipcode zones. The first three measures (intersection count, average streets per node, and average street length) reflected the number of intersections, the connectivity at intersections, and street length that could differentiate dense gridded city streets from fishbone-like rural networks. The fourth measure, average circuity, captured winding and looping roads common in suburban and rural areas. These four measures together should sufficiently characterize the nodes, edges, and paths in Lynch’s legibility of a city. We used points of interest to represent Lynch’s district and landmark elements. Different network measures and points of interests may be more or less useful in different modes of navigation by foot, car, or public transportation. Nevertheless, each navigation mode utilizes some external cues or landmarks in building mental maps and strengthens the mental maps through frequent commutes over time.

### Points of interest (POI) data

POI are locations people may find useful or interesting, hence, landmarks. Examples include skyscrapers, business establishments, monuments or open spaces, government or religious buildings, river junctures, bus stops, train stations, and many other natural or built structures. People commonly travel between and along POI, and POI’s visual or social prominence (such as water towers, gas stations, and supermarkets) naturally serve as landmarks for allocentric orientation and wayfinding.

The importance of POI for navigation is evident by more than 800 patents involving POI for navigation since 2002. Across the US, SafeGraph compiles, curates, and integrates over 6.4M shops, offices, schools, parks, hotels, train stations, and other places where people spend time or money. Individual POI (such as a city hall or a plaza) and POI agglomerates (such as a neighborhood) capture the idea of districts in Lynch’s five elements of city legibility.

We retrieved 831,049 POI in 27 categories from the SafeGraph Core Places (34) within the selected 154 3-digit zipcode zones. For simplicity, we reclassed the 27 categories into eight general classes (Table 3). All zipcode zones contained POI in all the eight general classes except for Zipcode 101 where no leisure POI was present. The total numbers of POI in each zipcode zone varied from 395 to 19,830 across all eight classes. POI density (the total number of POI divided by the total street length) in a zipcode zone varied from 3.8×10^−5^ to 0.41. The differential distributions of POI across the 154 zipcode zones were evident.

## Results and Analytic Approach

The data imposed two major constraints to traditional confirmatory factor analysis. First, NACC only provided geographic references of their subjects to 3-digit zipcode zones. An individual’s activities were commonly not confined in any zipcode zone, and those who lived on the edge of a zipcode zone might interact with the neighboring zipcode zone more than the residing one. Moreover, other known AD environmental factors could operate across zipcode zones, such as air pollutants (e.g., PM2.5). The high degree of spatial uncertainty could diminish any potential cognitive effects of environmental complexity from one’s zipcode zone. Second, street networks and POI were subject to change over time, so were the network measures and POI distributions. Therefore, the network and POI data likely mismatched what NACC subjects experienced during the annual surveys. The temporal uncertainty could further blur the cognitive effects of environmental complexity.

Thus, these sources of uncertainty deemed inferential statistics inappropriate since the data violated the basic assumptions of independence, normality, homogeneity, and linearity. In addition, the mixed scales of measurements and data distributions of network measures and POI counts invalidated the use of general linear regression models. Machine learning approaches are better positioned to account for non-linear relationships and noisy, messy high-dimensional data. Hence, instead of inferential statistics, we used category prediction of cognitive characterization for each 3-digit zipcode zone to elicit the potential connections between AD development and environmental complexity represented by network measures and POI distributions (i.e., types and frequencies). Category prediction has been applied to the prediction of coronary heart disease using risk factor categories (35) and the prediction of fMRI activities with thinking about arbitrary concrete nouns (36). Following the same analytical thinking, we applied category prediction to assess if we could use network measures and POI counts in each zipcode zone to predict the cognitive class of the zipcode zone based on the ratios of cognitive diagnoses on NACC subjects in each zipcode zone.

Our analysis included the following two steps: (1) categorized zipcode zones based on the similarity of cognitive-ratio distributions; (2) developed a neural network model to predict the cognitive category of each zipcode zone based on network measures and POI frequencies.

### Cognitive classification of zipcode zones

Based on NACC codes and descriptions, we characterized NACC subjects in five cognitive categories: Cognitively Normal, Impaired Not MCI (mild cognitive impairment), MCI, DEM (dementia) and AD (Alzheimer’s disease) and summarized the statistics in Table 1. The first four categories were exclusive and covered all NACC subjects in consideration. Since the number of NACC subjects varied across zipcode zones, we calculated the ratio of each category to the sum of the first four categories in each zipcode zone. The last UDS-based category (e.g., presumed AD etiology) included NACC subjects in MCI and DEM categories. Hence, the ratio of AD was based on the sum of MCI and DEM subjects in each zipcode zone. We applied agglomerative clustering with variance minimization to group the zipcode zones based on the five cognitive ratios at the last visits (i.e., the ratios of normal cognition, impaired but not MCI, MCI, and DEM, and the ratio of AD as a contributing factor to MCI or DEM). The grouping, if successful, supports the basis for categorical prediction (detailed in Neural Network Model section).

**Figure 2.**
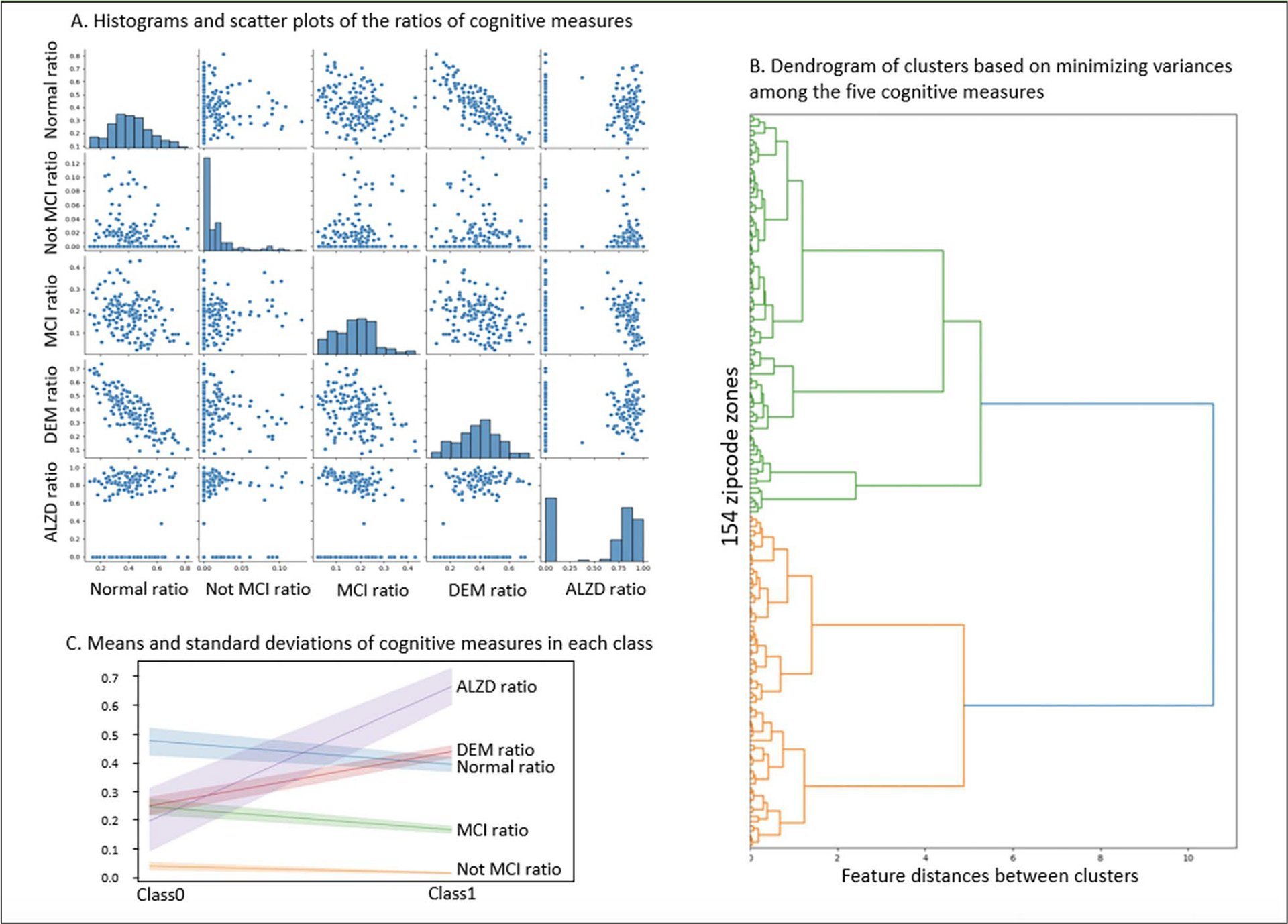
The selected cognitive measures across zipcode zones

Histograms indicated that Normal and DEM approximated normal distributions across zipcode zones. However, zipcode zones skewed towards very low Not MCI ratios, and the majority of zipcode zones were with MCI ratios less than 0.3. Moreover, zipcode zones showed either with very low (~0.0) or high (> 0.6) AD ratios in a bimodal distribution (Figure 2A). The scatter plots implied a negative correlation between Normal and DEM measures but no apparent correlations among the other cognitive measures. The dendrogram based on agglomerative clustering suggested that the five cognitive measures could separate the 154 zipcode zones into two classes (Figure 2B). Zipcode zones in Class0 were characterized with higher Normal and MCI ratios and lower DEM and AD ratios than those in Class1 (Figure 2C). Not MCI ratios remained low in both classes, yet displayed a slightly wider variance of MCI in Class0 than in Class1. Based on the distribution of cognitive measures, we named Class0, the Cognitively-Normal class, and Class1, the AD-inclined class.

The number of NACC subjects in the Cognitively-Normal class (Class0) were much smaller than the number in the AD-inclined class (Class1). However, potential confounding factors: Sex, Education, Age, and Race, to DEM and AD were comparable between the two classes (Tables 4 and 5). Both classes had more female than male subjects (9.4% more female in Class0, and 7.6% more female in Class1). The difference in sex distributions between the two classes was less than 2%. Education levels were also comparable between the two classes. NACC education encoded the number of years in education or with degree completions: 12 for high-school graduates or GED, 16 for college, 18 for master’s, 20 for doctorate, and 99 for unknown. Cognitively-Normal class had 0.75% (or 24) of subjects with education unknown, comparable to 0.69% (or 111) in AD-inclined class. For the rest of the subjects, male subjects had higher average numbers of years of education than female in both classes with around 16 years for male subjects and slightly less than 15 years for female subjects. All selected subjects had data on their birth years and comparable in both classes, but female subjects in the Cognitively-Normal class were on average 2 years younger than other selected subjects. Racial compositions of males and female subjects in Cognitively-Normal class were on par with AD-inclined class (Table 5). The ratios of different race groups were in proportion between the Cognitively-Normal and AD-inclined classes. In summary, subjects in the two classes of zipcode zones exhibited highly similar distributions of sex, age, race, and education years. These potential confounding factors were unlikely to contribute differences in cognitive ratios of NACC subjects in the two classes of zipcode zones.

**Figure 3.**
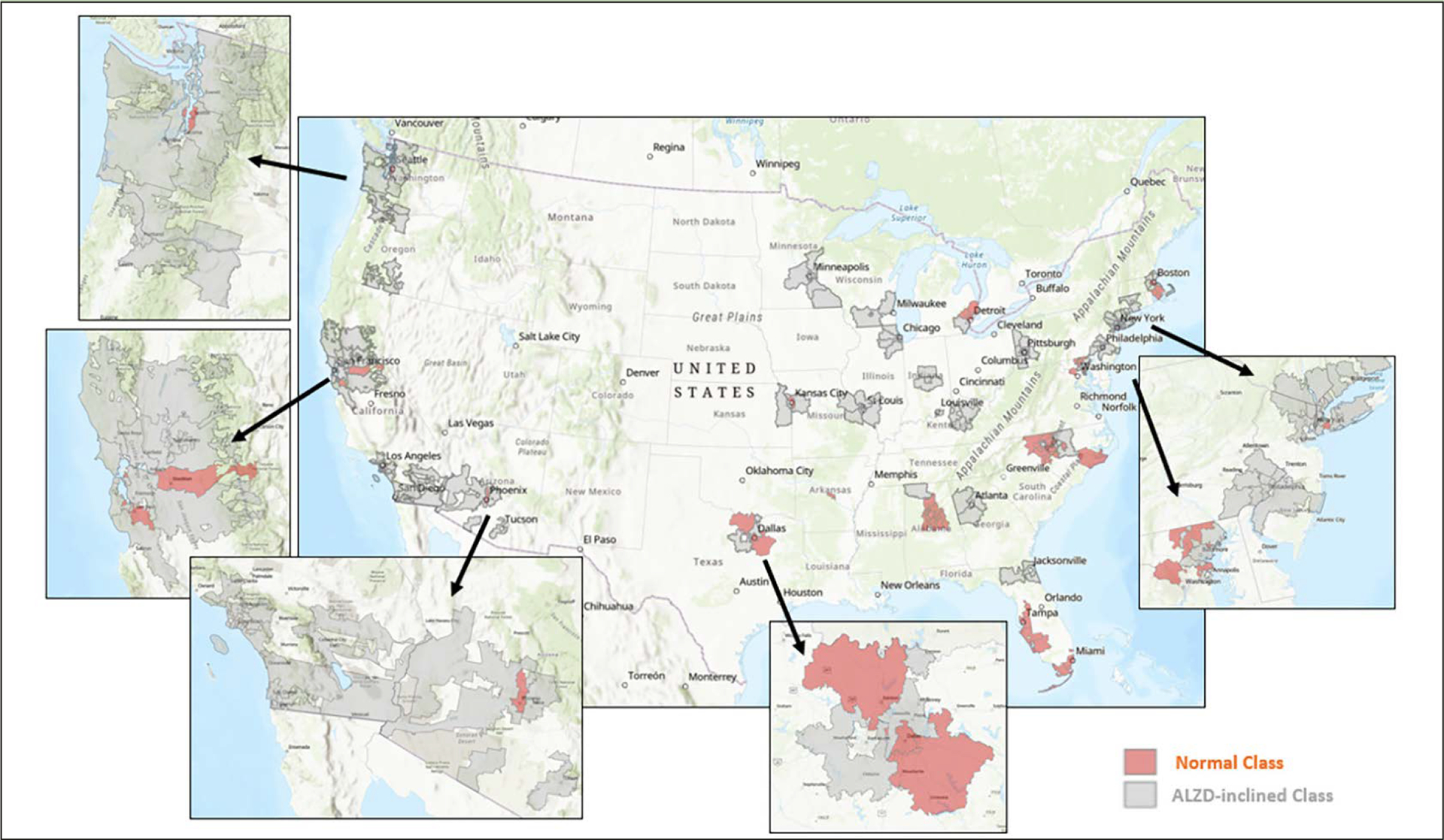
Cognitive classification of the selected 154 zipcode zones

Figure 3 displays the classification and geographic distribution of the selected 154 zipcode zones. The area coverage of each 3-digit zipcode zone varied significantly across the continental US. In general, urban zipcode zones were smaller than rural ones. The areal differentials of these zipcode zones could blur statistical patterns in the data. Nevertheless, zipcode zones in the AD-inclined class appeared more common in rural areas than in urban areas. Exceptions were around Pittsburg, Chicago, Minneapolis, St. Louis, and Los Angeles, for example. Likewise, the Cognitively-Normal class also included zipcode zones likely located in rural areas, such as the zones east and south of San Francisco and northwest and southeast of Dallas. Next, we calculated network measures and POI frequencies to represent environmental complexity in each zipcode zone, and then developed a neural network model to make categorical prediction of the cognitive class for each zipcode zone.

### A neural network model to predict the cognitive category of each zipcode zone

We developed a neural network model to make categorical predictions under the assumptions that the potential influence of environmental complexity on cognitive status was non-linear and with unknown latent factors. The predictors included the four network measures and eight POI types from Tables 2 and 3. The four network measures characterized edges, nodes, and curvatures of streets and the types of POI frequencies reflected both the variety and availability of landmarks. Together, these 12 input features represented the navigability of the correspondent zipcode zone (or in Lynch’s term, the legibility of a city).

**Figure 4.**
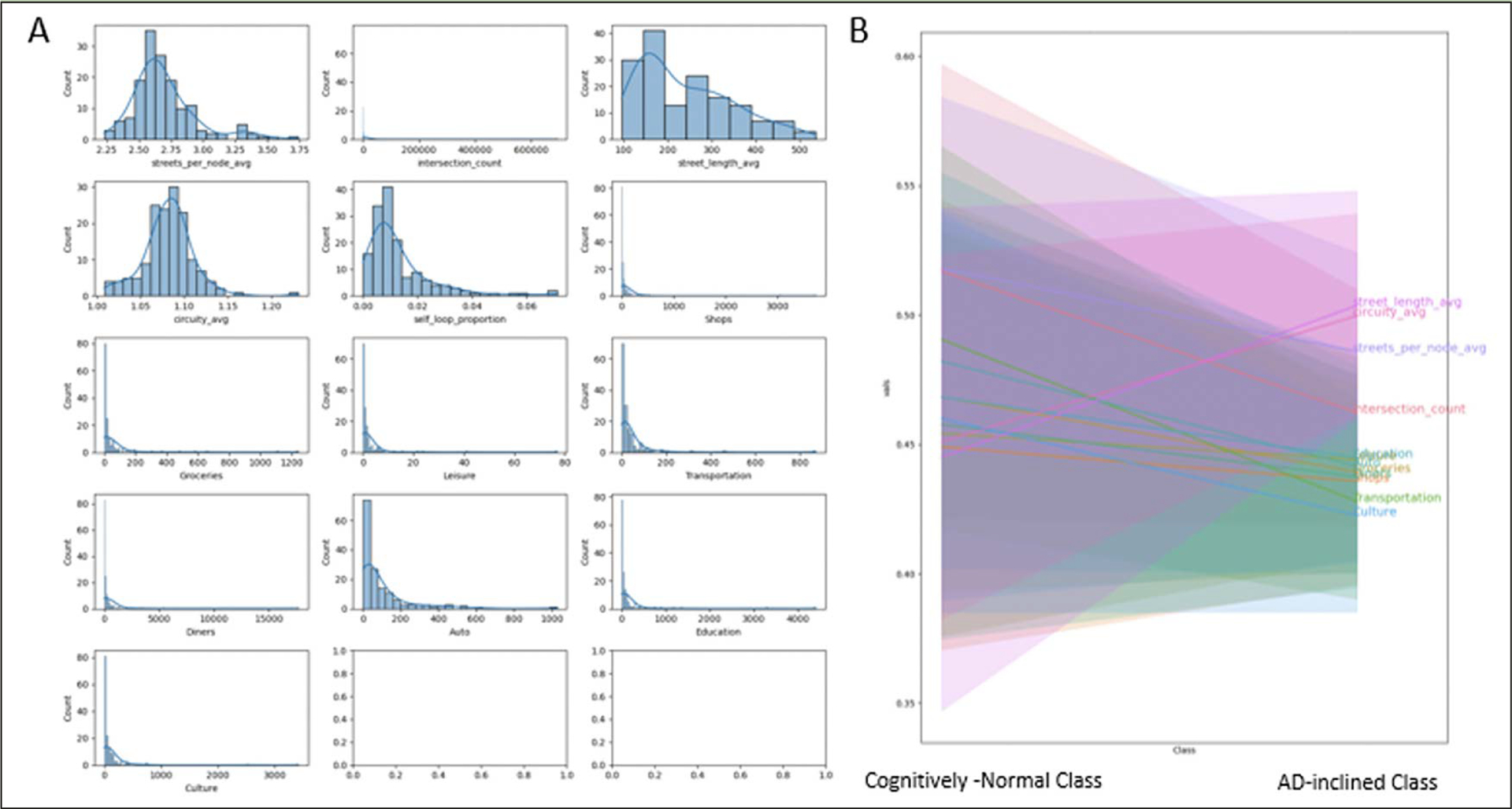
A: Frequency distributions of network measures and road-intersection and POI counts per 100 km of road length in respective zipcode zone, and B: The measures and count-density distributions in “Cognitively-Normal” and “AD-inclined” zipcode zones The histograms indicate that most of these data are skewed and required Yeo-Johnson transformation to preprocess the data for building the neural network model.

POI occurrences and network measures interplay within each zipcode zone. Shops, schools, and other POI are located along roads. Zipcode zones with longer total street lengths provide more opportunities for POI and intersections. Therefore, the study calculated POI and intersection counts per 100km street length (i.e., street-density counts) in individual zipcode zones. These POI and intersection street-density counts show highly skewed histograms across zipcode zones. Most zipcode zones have low POI counts, but outliers with high POI counts are apparent (Figure 4A). Yeo-Johnson transformation helped remedy data skewness to facilitate machine learning. We normalized POI counts and intersection counts by the total street length in each zipcode zone and applied Yeo-Johnson transformation to adjust for skewed frequency distributions. Figure 4B shows decreases in densities for all POI types and intersection counts and increases in street-length average and circuity average in AD-inclined class, while the opposite trends display in the Cognitively-Normal class.

Based on the dendrogram (Figure 2B), we experimented three neural network architectures, each with two hidden layers of 3 and 2 nodes, 4 and 2 nodes, and 5 and 2 nodes, respectively. We randomly selected 80% of the zipcode zones for training and reserved 20% for testing. We performed 5-fold cross-validation with the training data to identify the best architecture, activation functions for non-linear transformation, learning rate, and solver used to estimate the model parameters. Cross-validation analysis confirmed that the model with two hidden layers of 4 and 2 nodes with sigmoid activation functions, and Limited-memory Broyden-Fletcher-Goldfarb-Shanno (LBFGS) solver performed the best.

**Figure 5.**
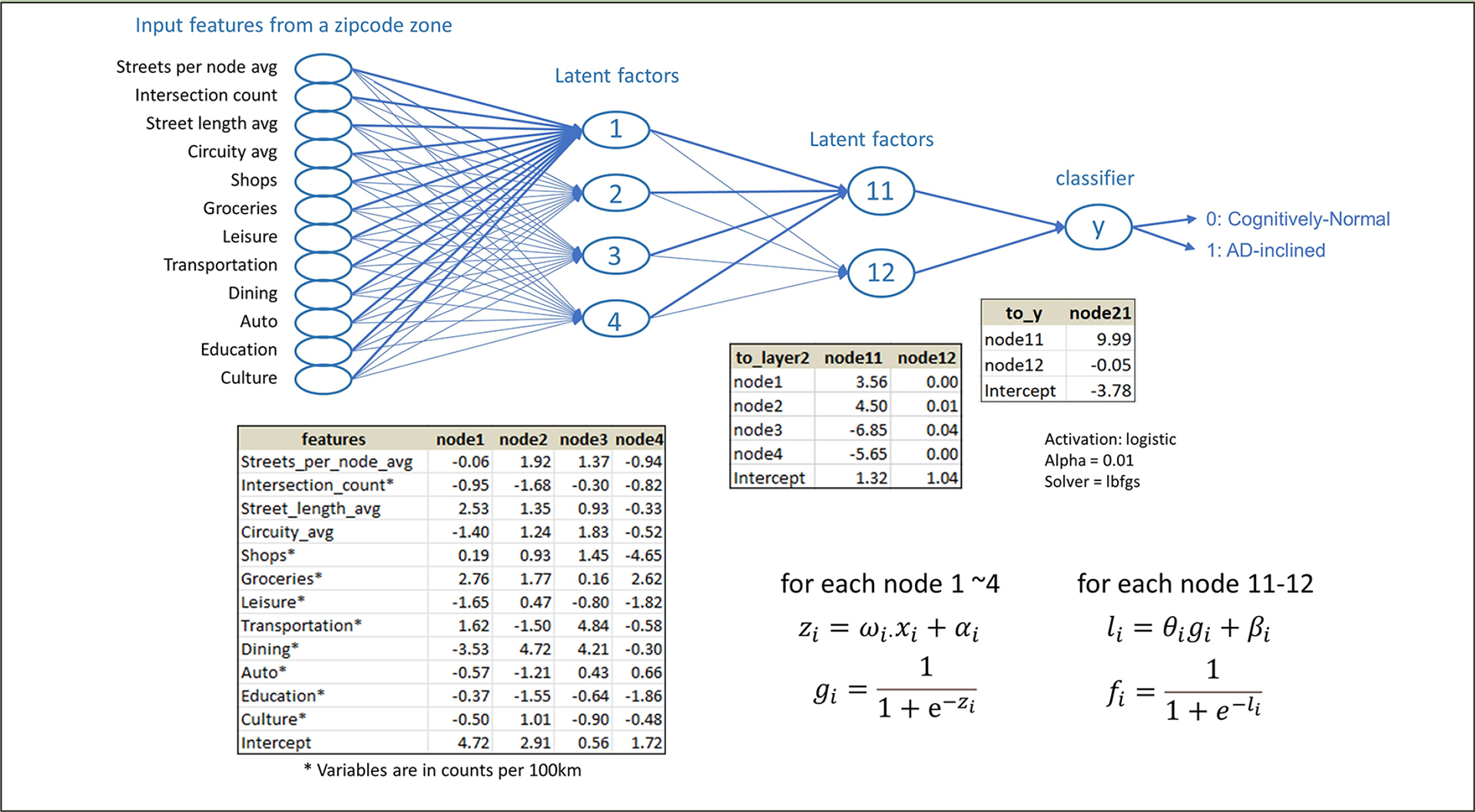
The neural network model built on the study data for categorical prediction of the cognitive status for zipcode zones

Whereas structural equation modeling would apply prior knowledge about the mechanistic relationships among independent variables to set latent factors, neural network modeling embeds all possible latent factors from input features in the model architecture. The best model in our experiment included four linear regressions from the input layer to the first hidden layer, four sigmoid transformations and two linear regressions from the first hidden layer to the second hidden layer, and two sigmoid transformations and a linear combination of the sigmoid transformations from the second hidden layer to the output that estimated the most likely class (Cognitively-Normal or AD-inclined) for the input zipcode zone (Figure 5).

**Figure 6.**
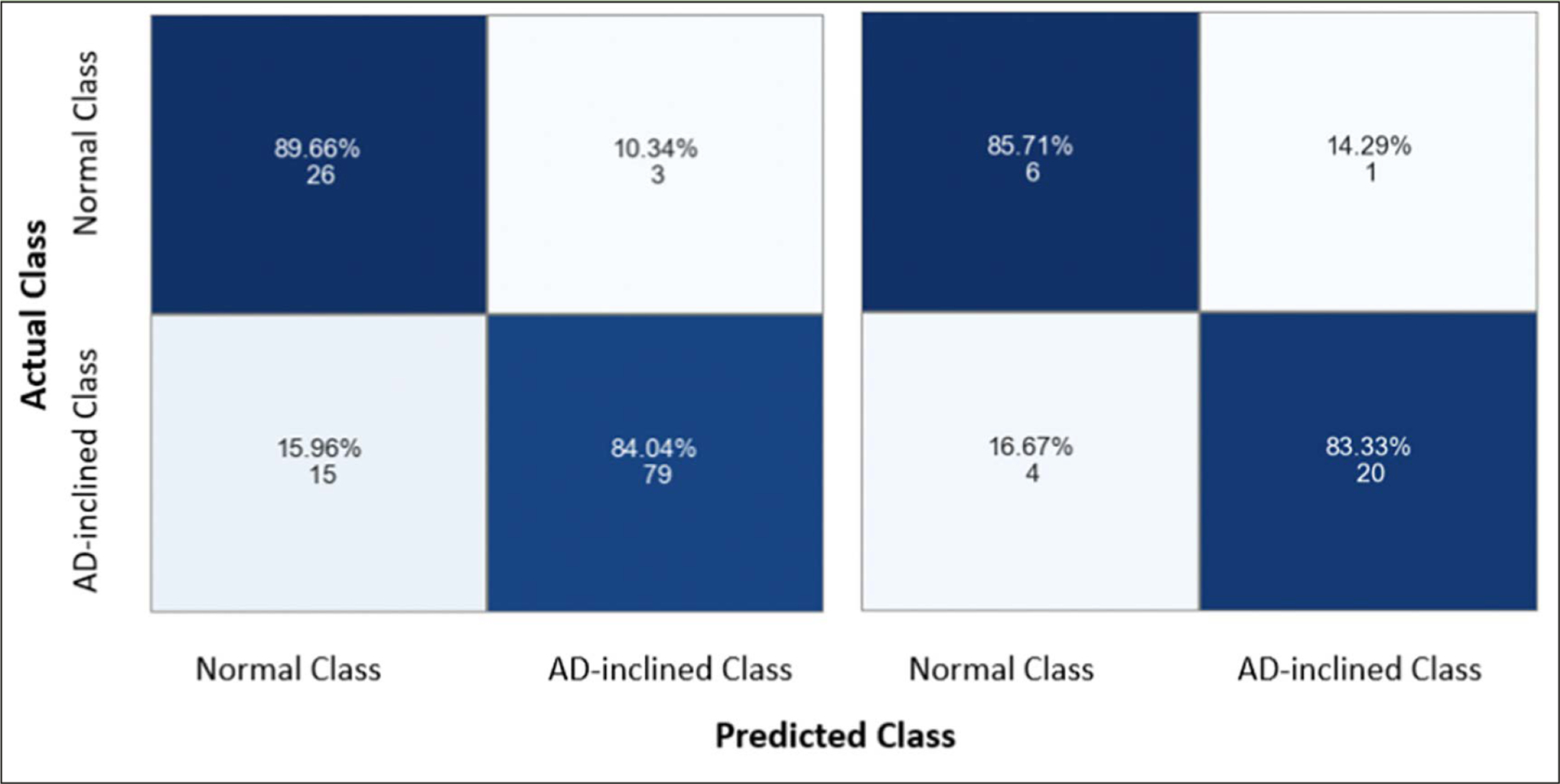
Confusion matrices for assessing model performance The model did not overfit the training data because it performed comparably well with the testing data. The model correctly predicted 89.66% and 84.04% zipcode zones in the Cognitively-Normal and AD-inclined classes, respectively, with training data (the left grid), and 86.71% and 83.33% with testing data (the right grid). The integers indicate the actual numbers of zipcode zones in each prediction quadrant from a total of 154 zipcode zones in the study.

As with all neural network models, the non-linear transformation complicates the interpretation of coefficients in the linear regressions and the marginal effects of a given input feature on predicting the output class. Instead, machine learning approaches emphasize assessing model performance. The neural network model performed well on categorical prediction on the testing data at 83.87% accuracy, 95.24% precision, 83.33% recall, and 0.8889 F1-score (i.e., the best F1-score equal to 1). The confusion matrices for training and testing data showed comparable results, suggesting that the model was not overfitting (Figure 6). With the training data, the model predicted correctly 89.66% zipcode zones in the Cognitively-Normal class and 84.04% zipcode zones in the AD-inclined class. However, the model mis-classified 10.34% zipcode zones in the Cognitively-Normal class and 15.96% zipcode zones in the AD-inclined class. Applying the model to the testing data that the model had not seen before, the model performed comparably well with correct predictions of 85.71% zipcode zones in the Cognitively-Normal class and 83.33% in the AD-inclined class. If environmental complexity (represented by network measures and POI) had no relevance to the classes that grouped zipcode zones based on cognitive differences in NACC subjects, a random chance would result in 50% for all four quadrants of the confusion matrix.

## Discussion

This exploratory retrospective study leveraged the large dataset collected in the NACC regarding participants’ diagnosis at last visit and demographic and environmental data computable from participant zipcode data. Our proposed neural network model demonstrates the high predictability of cognitive status classification (i.e., Cognitively-Normal from AD-inclined) based on network measures and POI-type frequencies in 3-digit zipcode zones. Our conceptual theory connects neural and environmental systems: navigating in a complex network-landmark system invokes cognitive demands on reading and mapping the physical space, which furthermore excites spatial cells in the hippocampal formation to build cognitive maps. The locations of nodes and POI may stimulate place cells. Edges, nodes, their topological connections, and navigating among them may fire grid cells. Besides place cells and grid cells, we also excite the head orientation cells and boundary cells when we use POI as allocentric landmarks to orient ourselves, traverse through the networks, visit POI, and perceive how changes in POI-types may reflect zoning changes as we travel through stores to schools, for example. If the theory holds, people living in a more complex environment tend to have more frequent activation of the spatial cells system and hence shall be less vulnerable to Alzheimer’s disease. Our findings support the theory as zipcode zones in the Cognitively-Normal Class are mostly located around major metropolitan areas and the AD-inclined zones tend to be rural areas.

A recent citizen-science study gamified wayfinding, path integration, and spatial working memory in a virtual reality, Sea Hero Quest, recruiting 3.9 million participants worldwide and concluded, among other conclusions, that rural or mixed city-rural participants (self-reported) exhibited better navigation abilities than participants from cities. Important factors that determine navigation difficulty were the number of decision points and the topological structure of the environment, and the game could successfully track at-genetic risk of AD participants over time (37). The study seemed to suggest that relatively simple rural environment might enhance one’s navigation abilities, but an alternative explanation could be the simulated sea environment may be more similar to rural than urban settings. Many studies have contrasted the prevalence and incidence of dementia and Alzheimer’s disease between rural and urban areas. Russ et al. (38) systematically reviewed 51 articles out of 12,580 publications extracted from 19 databases. The 51 articles came from 35 unique studies across the world but mostly in Europe, Canada, and the US. They synthesized qualitative findings and moreover performed a meta-analysis of 13 articles to draw quantitative summaries. Acknowledging the risk of bias within and across studies in sampling, diagnosis, and ambiguity in the definition of rurality, Russ et al. concluded a stronger effect of rural living on Alzheimer’s disease than other dementia types, with double the prevalence risk (see Table 6). Recent studies reiterate environmental or socioeconomic risk factors or limited healthcare access when interpreting the rural effect (25, 39, 40). In contrast, other studies suggest that environmental exposure to PM2.5, ozone, nitrogen dioxide, and nanoparticles increases the risk of Alzheimer’s disease among urban dwellers (23, 24, 41–43). Rural and urban settings and lifestyles can vary significantly in different countries. Therefore, generalized statements on rural and urban effects warrant further scrutiny.

To the best of our knowledge, our research is the first study to associate environmental complexity with dementia and Alzheimer’s, which opens a new perspective to uncover new factors driving the risk of rural living. Our premise posits that a complex environment increases the demand for cognitive map building, which enhances activities in the spatial cell system in the hippocampal formation and fosters health benefits to our spatial cognition and memory. Hence, such beneficial mediation of cognitive map building may serve as a non-pharmaceutical intervention to delay onset or slow the progression of dementia, and particularly Alzheimer’s disease.

### Limitations

The finest-grained spatial reference available to us for NACC subjects is 3-digit zipcode zones, which are significantly smaller in urban than rural areas (Figure 3). The selected 154 zipcode zones have spatial extents ranging from less than 2 to more than 35,000 km^2^. Such coarse and varying area units prevent any detailed interrogation of environmental complexity on individual NACC participants for three main reasons: high spatial heterogeneity within large zipcode zones, participant’s activity space that could be limited to smaller areas within a large zipcode zone or could go beyond a small zipcode zone, and participants who might have AD elsewhere before moving into the zipcode zone recorded in NACC data. Moreover, uneven POI distributions, especially in a large zipcode zone, can over-estimate POI effects. For example, popular strip malls house high-density clusters of shops and dining in suburban areas but only provide single or limited landmarks for navigation purposes.

Even though the POI density and intersection density variances along street networks appear high, the findings remain aligning with our environmental complexity theory. The greater street-length average and circuity average in AD-inclined zipcode zones also suggest lesser environmental complexity in these zones. As a limitation to these data as currently available, our modeling suffers from (1) the coarse spatial unit of analysis (i.e., 3-digit zipcode zones) and (2) substantial data heterogeneity. The two data issues prevail in all three independent data sources: NACC subjects, OpenStreetMap, and Safegraph POI records, and in spatial aggregation to zipcode zones. Despite these two major data limitations, the neural network model is still able to make categorical predictions on the testing data with high accuracy, precision, and F1-score metrics.

## Conclusion

Our research aimed to elicit potential environmental-neural connections to dementia and Alzheimer’s diseases. We uniquely combined interdisciplinary fields to ground our predictions with major theories from both the geospatial and cognitive neuroscience fields. We grounded the study with the theory that frequent stimulations to our brain’s spatial cell system can improve the development of the hippocampal formation and subsequently lead to beneficial effects on reducing dementia and Alzheimer’s diseases. We followed Lynch’s theory on urban legibility for navigability and selected four network measures and eight POI categories to capture routing options and landmarks, corresponding to firing activities of place cells, grid cells, head orientation cells, and boundary cells. Our input data exhibited high degrees of heterogeneity, skewness, and multiple measurement scales, making application of inferential statistics inappropriate. Instead, we developed a neural network model that ingests network measures from OpenStreetMap and counts of POI types from Safegraph records to predict the cognitive categories of zipcode zones based on NACC data. Even with non-linear effects of network measures and POI on spatial cognition, our proposed neural network model performed very well, predicting cognitive categories of zipcode zones with the selected four network measures (Table 2) and counts of eight POI types (Table 3), achieving 83.87% accuracy, 95.24% precision, 83.33% recall, and 0.8889 F1-score on testing data.

Our findings showed AD-inclined zipcode zones contained longer street-length average, higher circuity, and slightly lower POI counts. Most of the Cognitively-Normal zipcode zones appeared in or near urban areas. However, many major cities, such as Chicago and Los Angeles, had more similar cognitive ratios among NACC subjects to those in the AD-inclined class. While the study suffered high degrees of data uncertainty, the model’s high classification predictability nonetheless gave strong implications of the potential environmental-neural connections and warrants prospective human-subjects research to control confounding factors and examine the effects of environmental complexity on the aging brain, dementia and Alzheimer’s disease. If the theory holds, cognitive map building may serve as an intervention to dementia or AD, and can guide human factors research, such as redesigning GPS navigation systems to drive cognitive map building, rather than diminishing the opportunities with more mindless navigation.

There is a growing number of studies affirming the higher risk of dementia and AD on rural residents, but these are all associative in nature. The emerging field of environmental neuroscience seeks causality between environmental exposome and neurological responses with foci on toxicants’ effects on neural systems and gene-environment interactions (44). Our research suggests that, beyond the conventional behavioral risk factors, such as smoking, diet, and exercise, spatial navigation strategy can also be a modifiable risk factor for dementia and AD. Effective spatial navigation strategies, like being aware of routes and landmarks during daily commutes or running errands, facilitate cognitive map building and stimulate space cell systems. This study demonstrates the impressively high predictability of cognitive categories of zipcode zones based on environmental complexity features, but the input data in this exploratory study do not allow us to establish any statistical effects of environmental complexity on dementia and AD. Many studies asserted spatial navigation deficits as a biomarker for preclinical AD with controlled experiments (10, 11, 45, 46). A recent experiment showed cognitive mapping and route learning tasks evidenced strong diagnostic accuracy of longitudinal clinical AD decline (47). A carefully designed human subject experiment on the effects of environmental complexity (a potential intervention variable) on cognitive mapping, route learning and ensuing dementia and AD progression is deemed as the necessary next step and warranted by the current study findings.

## Figures and Tables

**Table 1.** NACC subjects with multiple visits and stayed in the same zipcode zones

Cognitive diagnosis at last visit	NACC data codes	N selected for study (154 zipcode zones)	N total in NACC data (682 zipcode zones)
1. Normal cognition	NACCUDSD = 1	8,081	9,285
2. Cognitive impaired but did not meet the criteria for MCI	NACCUDSD = 2	353	406
3. Either amnestic or non-amnestic MCI at the last visit	NACCUDSD = 3	3,548	4,117
4. With a diagnosis of dementia	NACCUDSD = 4	7,212	8,745
5. Presumed Alzheimer’s disease as the etiological diagnosis for any cognitive impairment (dementia, MCI, or impaired not MCI)	NACCALZP = 1	7,455	10,727

**Table 2.** Network measures in each selected zipcode zone

Measure	Definition
1. Intersection count	Number of intersections in networks
2. Average streets per node	Mean number of physical streets that emanate from each node (intersections and dead-ends)
3. Average street length	Mean edge length in the undirected representation of network (meters)
4. Average circuity	Total edge length divided by the sum of great circle distances between the nodes incident to each edge

**Table 3.** Points of Interest (POI) classes

General Class	Top POI categories in SafeGraph database
Automobile	Automobile Dealers, Automotive Equipment Rental and Leasing, Automotive Parts, Accessories, and Tire Stores, Automotive Repair and Maintenance
Cultural	Museums, Historical Sites, and Similar Institutions, Religious Organizations
Dining	Bakeries and Tortilla Manufacturing, Restaurants and Other Eating Places
Education	Colleges, Universities, and Professional Schools, Elementary and Secondary Schools, Junior Colleges, Child Day Care Services
Groceries	Grocery Stores, Grocery and Related Product Merchant Wholesalers
Leisure	Amusement Parks and Arcades, Gambling Industries, Greenhouse, Nursery, and Floriculture Production
Shop	Apparel, Piece Goods, and Notions Merchant Wholesalers, Book Stores and News Dealers, Clothing Stores, Department Stores
Transportation	Gasoline Stations, Interurban and Rural Bus Transportation, Rail Transportation, Postal Service

**Table 4.** Distributions of sex, education year, and age in Cognitively-Normal and AD-inclined classes of zipcode zones

		Sex		Education		Birth year	
		Counts	ratio	mean (years)	std (years)	mean	Std (years)
Cognitively- Normal	Male	1287	40.29%	16.14	3.10	1937	10.39
	Female	1907	59.71%	14.97	3.15	1939	10.38
AD-inclined	Male	6588	41.18%	15.95	3.36	1937	11.12
	Female	9412	58.83%	14.79	3.38	1937	11.56

**Table 5.** Racial compositions within sexes in each class of zipcode zones

	Male				Female			
	Cognitively-Normal		AD-inclined		Cognitively-Normal		AD-inclined	
	Counts	Percentage	Counts	Percentage	Counts	Percentage	Counts	Percentage
White	1097	85.24%	1385	85.97%	5664	72.63%	7035	74.75%
Black	149	11.58%	455	9.24%	609	23.86%	1812	19.25%
Native	5	0.39%	13	0.30%	20	0.68%	45	0.48%
Hawaiian	0	0.00%	1	0.06%	4	0.05%	7	0.07%
Asian	32	2.49%	42	2.88%	190	2.20%	263	2.79%
Other	3	0.23%	6	1.15%	76	0.31%	200	2.12%
Unknown	1	0.08%	5	0.38%	25	0.26%	50	0.53%
Total	1287	100.00%	1907	100.00%	6588	100.00%	9412	100.00%

Note. Black: Black or African American; Native: American Indian or Alaskan Native; Hawaiian: Native Hawaiian or other Pacific Islanders

**Table 6.** Effects of rural living based on a meta-analysis by Russ et al. (2012)

		90% confidence interval
Dementia	Prevalence Odds ratio = 1.11	0.79–1.57
	Incidence Odds ratio = 1.20	0.84–1.71
Alzheimer’s Disease	Prevalence Odds ratio = 2.22	1.19–4.16
	Incidence Odds ratio = 1.64	1.08–2.50

## Data Availability

The data that support the findings of this study are available from the National Alzheimer’s Coordinating Center (https://naccdata.org) but restrictions apply to the availability of these data, which were used under formal approval for the current study, and so are not publicly available from the authors.
